# Biodegradable, lignin-based encapsulation enables delivery of *Trichoderma reesei* with programmed enzymatic release against grapevine trunk diseases

**DOI:** 10.1016/j.mtbio.2020.100061

**Published:** 2020-06-20

**Authors:** S. Peil, S.J. Beckers, J. Fischer, F.R. Wurm

**Affiliations:** a Max-Planck-Insitute for Polymer Research, Ackermannweg 10, 55128, Mainz, Germany; b Institute for Biotechnology and Drug Research, Erwin-Schrödinger-Str. 56, 67663, Kaiserslautern, Germany

**Keywords:** Agriculture, *Trichoderma*, IBWF 034-05, Plant protection, Layer-by-layer, Esca, Biopolymer

## Abstract

Antagonistic fungi such as *Trichoderma reesei* are promising alternatives to conventional fungicides in agriculture. This is especially true for worldwide occurring grapevine trunk diseases, causing losses of US$1.5 billion every year, at which conventional fungicides are mostly ineffective or prohibited by law. Yet, applications of *Trichoderma* against grapevine trunk diseases are limited to preventive measures, suffer from poor shelf life, or uncontrolled germination. Therefore, we developed a mild and spore-compatible layer-by-layer assembly to encapsulate spores of a new mycoparasitic strain of *T. reesei* IBWF 034-05 in a bio-based and biodegradable lignin shell. The encapsulation inhibits undesired premature germination and enables the application as an aqueous dispersion via trunk injection. First injected into a plant, the spores remain in a resting state. Second, when lignin-degrading fungi infect the plant, enzymatic degradation of the shell occurs and germination is selectively triggered by the pathogenic fungi itself, which was proven *in vitro*. Germinated *Trichoderma* antagonizes the fungal pathogens and finally supplants them from the plant. This concept enables *Trichoderma* spores for curative treatment of esca, one of the most infective grapevine trunk diseases worldwide.

## Highlights

• First report on an enzyme-responsive drug delivery system for fungal spores.
• Layer-by-layer encapsulation provides aqueous, surfactant-free spore dispersion.
• Curative treatment of grapevine trunk diseases with *Trichoderma reesei*.
• Lignin shell induces a resting state of *T. reesei* spores.
• Pathogens trigger the enzymatic lignin degradation and selective spore germination.

## Graphical abstract

**Figure:**
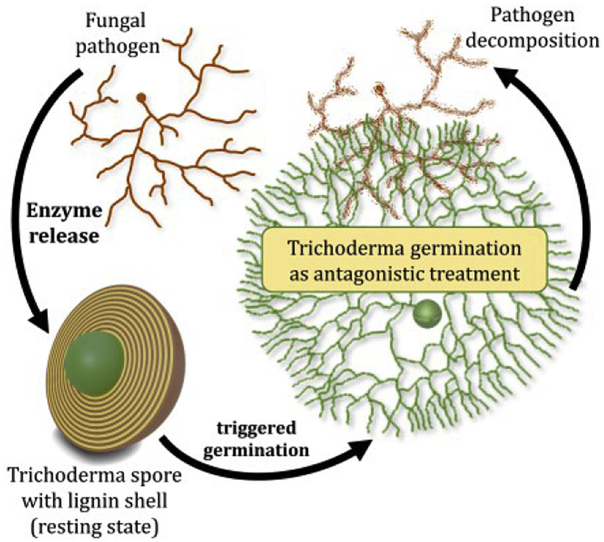

## 1 Introduction

The use of biological control agents (BCAs) in plant protection offers great potential for a sustainable future in agriculture. BCAs are naturally occurring organisms, including bacteria and fungi, which are natural enemies of plant pathogens and might replace chemical drugs in several applications [1,2]. In recent years, fungi of the genus *Trichoderma* have received increased attention because of their antagonistic potential against a wide range of fungal diseases [[3], [4], [5]]. *Trichoderma*, compared to most fungal pathogens, shows faster metabolic rates, the formation of antimicrobial metabolites, and the ability of mycoparasitism [6], which results in blocking of the given habitat for other pathogenic microorganisms. Afterward, *Trichoderma* lives in symbiosis with the host plant. In comparison to conventional pesticides, which often lack selectivity, might accumulate in the environment, or have toxic side-effects on biodiversity and human health, none of these undesirable effects are known for the use of *Trichoderma* strains. In addition, *Trichoderma* lives in symbiosis with the host plant, as secondary metabolites trigger the internal defense system of the plant whereby resistance against future pathogens is increased [[7], [8], [9]]. However, a wide-spread application has not been possible to date because of the limited shelf life of *Trichoderma* formulations or uncontrolled germination of the spores during storage time [10].

Herein, we present the first aqueous formulation of *Trichoderma* spores that enable controlled germination and long-term dispersion stability by a mild layer-by-layer (LbL) deposition of biodegradable lignin derivatives [[11], [12], [13], [14], [15], [16]]. The deposited layers form a continuous shell that prevents spore germination and can be opened by selective fungal degradation causing the release of the active spores in case of an esca infection. Mycoparasitism combined with controlled germination and selective release enables the spores to act as biofungicides against plant diseases impossible to treat with conventional pesticides.

Especially the worldwide occurring fungal disease esca, one of the most infective grapevine trunk diseases, is a well-known example of the failure of the conventional pesticide application [17]. Spores of pathogenic fungi enter the plant mainly via pruning wounds and the pathogen grows inside the grapevine trunk. Once a plant is infected, esca cannot be cured because the pathogens' location inside the grapevine trunk cannot reached by conventional spraying of fungicides [18]. Up to date, all commercially available treatments are based on preventive measures, including permanent disinfection of pruning tools, protection of wounds on the vine, and repetitive preventive spraying of fungicides in high doses [17]. Once a plant is infected, the pathogens segregate lignin-degrading enzymes (e.g. laccases and peroxidases) [19], which lead to trunk wood decay and withering of the plant. For progressed infections, the entire grapevine needs to be replaced [20]. To prevent further spreading, removal and burning of the infected material are essential. Particularly after the ban of sodium arsenite in Europe in 2003 [21], because of its carcinogenicity [22,23], the lack of effective curative treatments against grapevine trunk diseases represents a big threat to the global wine industry, causing an estimated financial loss of US$1.5 billion/year [24]. Owing to its complex structure–property relationships, only a few applications for controlled release–based lignin are known [25,26]. We recently presented the first curative approach against esca, using lignin nanocarriers loaded with chemical fungicides, which were injected directly into the trunk of infected grapevine plants [[27], [28], [29], [30]]. Moreover, an application of *Trichoderma* against esca has been investigated but is limited to preventive measures, protecting only pathogen-free vines in nurseries and young vineyards up to 4 years [31,32]. Up to date, no BCA has been reported as a curative treatment against esca.

**Fig. 1:**
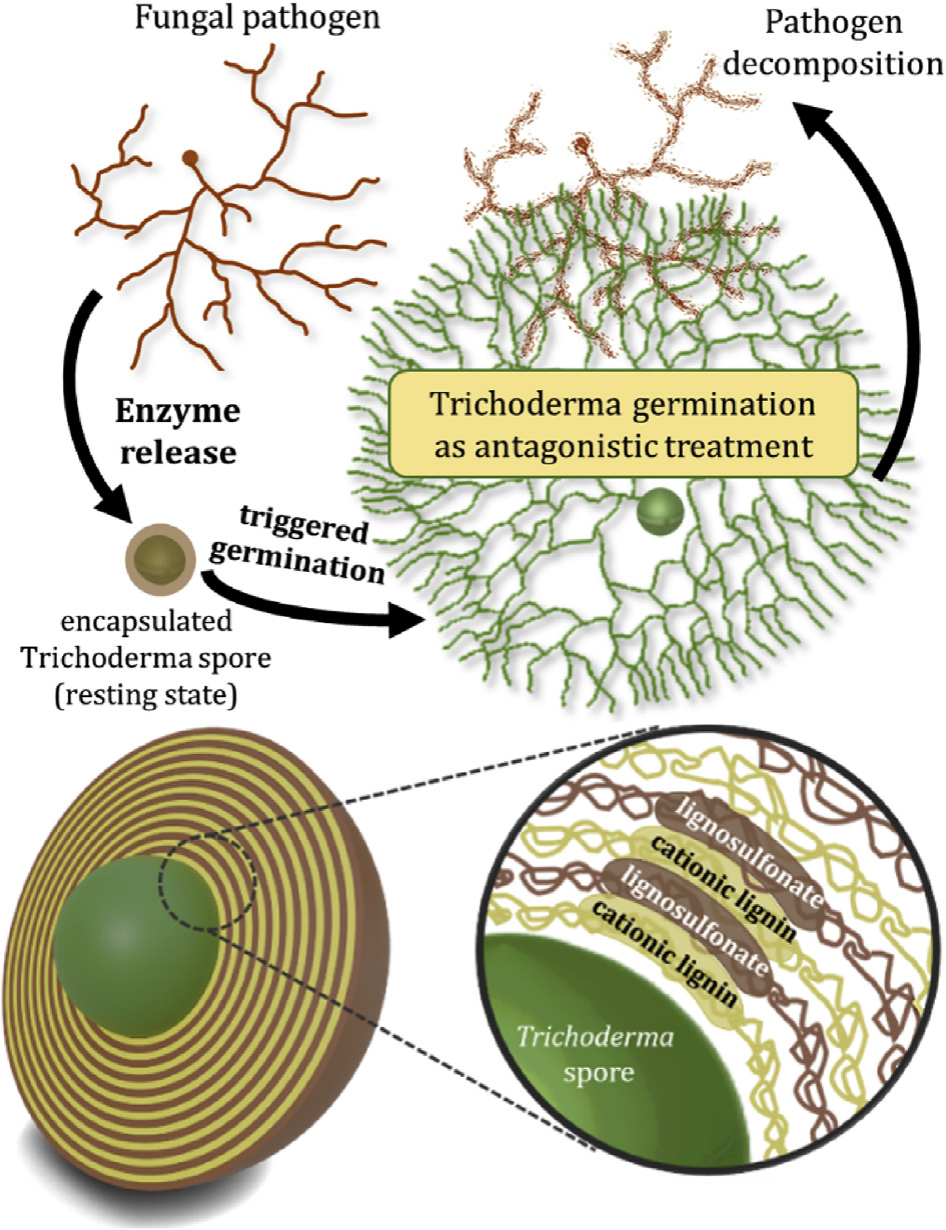
The concept for the delivery of *Trichoderma* spores as a biological control agent.

To the best of our knowledge, this is the first report on a *Trichoderma*-based delivery system with an enzymatic release for curative treatment of grapevine trunk diseases (Fig. 1). The herein developed spore-compatible LbL protocol produces an aqueous spore dispersion, which is applicable for the trunk injection technique and allows safe and convenient handling. Germination of encapsulated *Trichoderma* in liquid dispersion can be precisely controlled by the number of assembled lignin layers. Lignin-degrading enzymes, secreted by the pathogenic fungi itself, trigger the targeted enzymatic spore release. *In vitro* tests proved that the release leads to *Trichoderma* germination, which antagonizes the pathogenic fungi and finally supplants the pathogen. The application of the introduced *Trichoderma* formulation would lead to improved plant health and could reduce the number of fungicides in viticulture. Furthermore, the reduction of chemical fungicides would lead to beneficial effects on biodiversity and human health.

## 2 Materials and methods

### 2.1 Materials

All following solvents and chemicals were used without further purification: Agar (Sigma-Aldrich); ethanol (technical grade, Sigma-Aldrich); deuterium oxide (99.9 atom % D, Sigma-Aldrich); d-glucose (Sigma-Aldrich); glycidyltrimethylammonium chloride (technical ≥ 90 % based on dry substance, contains 2-–4 % chlorohydrin and 20-25 % water, Sigma-Aldrich); epoxy embedding medium (FLUKA); Kraft lignin (Lignin, alkali, Lot: #MKBV5831V, Sigma Sigma-Aldrich); malt extract (Sigma-Aldrich); osmium tetroxide solution (4 % in H_2_O, Sigma-Aldrich); paraformaldehyde (4 % in phosphate buffered saline (PBS), Alfa Aesar); poly(diallyldimethylammonium chloride) solution (20 wt% in H_2_O, average); poly(styrene sulfonic acid) sodium salt ($M_{\text{w}}=5\cdot 10^{5}\text{g}/\text{mol}$, Alfa Aesar); sodium hydroxide (TCI); sodium ligninsulfonate (TCI, product:#L0098, Lot: #V5VJF-NC); water (sterile, pyrogen-free, Ampuwa Plastipur, Fresenius Kabi); yeast extract (Carl Roth); petri dishes (90 mm, Carl Roth); bacterial cell spreaders (Carl Roth); micropipette (Eppendorf); Eppendorf safe lock tubes (1.5 mL, Eppendorf); Greiner centrifuge tubes (40 mL and 15 mL, Sigma-Aldrich); Miracloth filters (Merck Millipore).

### 2.2 Instruments and characterization techniques

For sterilization, a Hiclave HG-50 autoclave was used. Solutions and items such as Eppendorf safe lock tubes were autoclaved for 40 min at 121 °C. Solid surfaces were disinfected with 70 vol% ethanol solution in distilled water. For the creation of an aseptic work space, a Bunsen burner equipped with mobile gas cartridges was applied. For the LbL process, a microcentrifuge by Thermo Scientific (Heraeus Fresco 21) was used. A vortex shaker by IKA (VORTEX 3) was used at level 5 for redispersion afterward. In addition, a VWR ultrasound cleaning bath USC 1200 TH was used. Optical microscopy was performed at a Zeiss Axiophot equipped with a Leica DFC295 camera. Secondary electron microscopy images were taken with a Hitachi SU8400. Therefore, 5 μL of a spore dispersion consisting of roughly $10\cdot 10^{6}$ spores/mL were deposited on an Si wafer. The solvent was evaporated at room temperature overnight. For transmission electron microscopy (TEM) and energy-dispersive X-ray spectroscopy (EDX), a Zeiss EM912 was used. Therefore, spores embedded in an epoxy matrix were cut into thin slices of roughly 100 nm by the aid of a Leica EM UC7 microtome. The obtained histological sections were used for TEM and EDX analyses. Zeta potential measurements were carried out with a Malvern Zetasizer Nano Z instrument. Therefore, roughly $10\cdot 10^{6}$ spores in 0.8 mL sterile water were placed in the Zetasizer cuvette. Smoluchowski approximation was chosen for the calculation of the zeta potential from the obtained velocity data. Fourier-transformed infrared spectroscopy (FT-IR) spectroscopy was performed with a Perkin Elmer Spectrum BX in conjugation with attenuated total reflection technique. Nuclear magnetic resonance (NMR) spectroscopy was performed on a 300 MHz Bruker Avance system at 298.3 K. The measured spectra were processed with MestReNovaed 12.0.1 software (Mestrelab Research S.L.).

### 2.3 Growth of *Trichoderma reesei* IBWF 034-05 and antagonistic test assays

*Trichoderma* spores were inoculated on solid culture medium for 1 week until the whole petri dish was fully covered with spore-producing mycelium. Sterile water in an amount of 10–15 mL was added on the plate and a sterile bacteria spreader was used to disperse the spores in the liquid phase. Afterward, the spore dispersion was filtered with a Miracloth filter. The filtrate was collected in a sterile 40 mL centrifuge tube. After the spore concentration was determined via a Neubauer counting chamber, a defined number of spores (typically $500\cdot 10^{6}$) was placed in sterile centrifuge tubes and centrifuged at 5,000 rpm (2,348 rcf). After removal of the supernatant, spores were stored at 4 °C in a fridge.

Antagonistic efficiency of *T. reesei* against *Phaeomoniella chlamydospora* (*Pch*) and *Phaeoacremonium minimum* (*Pmi*) was determined *in vitro* by a confrontation assay. Agar disks with a diameter of 5 mm covered with fungi were taken from 14-day-old cultures of *Pch*, *Pmi*, and *T. reesei*, respectively. Disks of pathogens and *Trichoderma* fungi were placed on the opposite side of petri dishes containing yeast malt glucose (YMG) medium. The dual cultures in petri dishes were incubated for 5 days at 25 °C and three replicates were used in each test. Growth reduction of *Pch* and *Pmi* were determined optically by measuring the covered plate areas. To study hyphal parasitism of *T. reesei* for mycoparasitic activity against *Pch* and *Pmi*, the following method was used: small blocks of agar with fungal colonies were placed 55 mm apart on cellophane over YMG. The plates were inoculated at 25 °C. Colonies met approximately 3 days after inoculation. Small squares of cellophane were cut from the area of mixed growth, stained with cotton blue, and examined under a light microscope.

### 2.4 Spore quantification via Neubauer counting chamber

For the determination of the spore concentration, the original sample was diluted to a concentration of approximately $1-3\cdot 10^{6}$ spores/mL. A volume of 7 μL of this spore dispersion were placed into the Neubauer counting chamber. Afterward, the spores in four big squares of the counting grid were counted and the concentration of the original sample *c* in spores per milliliter was calculated by Eq. (1).

(1) $$c=\frac{N\cdot 10\;000}{N_{\text{sq}}}\cdot d$$

where, N is the number of counted spores, $N_{\text{sq}}$ is the number of counted big squares, and d is the dilution factor of the dispersion. The volume factor is 10,000 because one central square of the grid is layered with a volume of 1 mm^3^.

### 2.5 Synthesis of cationic Kraft lignin

To prepare cationic Kraft lignin, a modified synthesis procedure of Kong et al. [33] was applied. The OH group content in the used Kraft lignin batch was determined by a derivatization technique with 1,3,2-dioxaphospholanyl derivatives and ^31^P NMR, as previously described in the literature [34]. The analysis revealed an amount of 6.13 mmol of OH groups per gram. According to this number, the stoichiometric calculation was referred. One gram of Kraft lignin was dissolved in 99 g of 0.2 M NaOH. According to a molar ratio of 1:2 between OH groups and glycidyl trimethylammonium chloride (GTAC), 2.58 g of GTAC were added and the solution was continuously heated at 70 °C and stirred at 100 rpm for 1 h. The reaction solution was neutralized with 10 wt% sulfuric acid, while a brown precipitate occurred. The reaction mixture was dialyzed against distilled water for 3 days (molecular weight cutoff of the dialysis membrane was 1,000 Da); the water was changed twice a day. As the dialyzed product contained a water-soluble and a water-insoluble fraction, an aqueous extraction was added to collect water-soluble cationic lignin. Therefore, the product was washed thrice in 300 mL distilled water until the supernatant after centrifugation did not show any brown color. The water of the collected supernatant, which contained only the water-soluble fraction, was evaporated until a volume of approximately 10 mL remained. 0.5 g of water-soluble cationic lignin was obtained by freeze-drying. The reaction product was soluble up to approximately 0.25 wt% in sterile water at pH 6.

### 2.6 LbL encapsulation of *Trichoderma* spores

For a typical LbL encapsulation, 50 mL of the respective polyelectrolyte solutions (c=0.2 wt% for polycations as well as polyanions) were prepared and autoclaved afterward. *Trichoderma* spores were dispersed in sterile water with a concentration of $40-50\cdot 10^{6}$ spores/mL. One milliliter of this dispersion was filled in a 1.5 mL Eppendorf safe lock tube and centrifuged for a first washing step (3 min, 5,000 rpm [2,348 rcf]). During the removal of the supernatant with a 1 mL Eppendorf pipette, a very small fraction of around 100 μL water remained in the vessel to minimize the loss of spores. Afterward, 500  μL sterile water were added and the spores were redispersed (30 s of vortex shaking followed by 3 min ultrasonic bath). Then, 500 μL of the polycation solution were added and the sample was vortexed for 5 s. Subsequently, a waiting time of 15 min without agitation allowed the adsorption of polymer to the spore surface. After an additional centrifugation and redispersion cycle, 500  μL of the polyanion were added and incubated for 15 min. This procedure was repeated until the desired number of polyelectrolyte layers were obtained. After each redispersion in sterile water, the zeta potential of the spore dispersion was measured.

### 2.7 Cell wall staining by osmium tetroxide and embedding in an epoxy matrix

For the staining of the *Trichoderma* cell wall, three droplets of an aqueous 4 wt% osmium tetroxide solution were added to 1 mL of a spore dispersion, which contained $80\cdot 10^{6}$ spores. After 15 min, the sample was centrifuged and washed twice with sterile water. A LbL encapsulation as described earlier was carried out afterward. After 20 layers, the spores were centrifuged and redispersed in a 4% paraformaldehyde solution twice. The time for fixation was 30 min, respectively. After that, the solvent was exchanged. Therefore, the spores were washed successively with aqueous solutions of ethanol with concentrations of 30%, 50%, 70%, 90%, and 100% (twice). Subsequently, washing steps with solutions of epoxy embedding medium in ethanol with concentrations of 33%, 50%, 66%, and 100% (thrice) were added. In every washing step, the sample was given 10 min for sufficient solvent exchange. Owing to the viscosity of the epoxy embedding medium, centrifugation for the last three washing steps was performed at 9,000 rpm for 20 min instead of 5,000 rpm for 3 min, which was applied for the other washing steps. After the spores were embedded in epoxy medium, the sample was placed in an oven at 50 °C overnight for the curing reaction.

### 2.8 Preparation of YMG medium

YMG medium for the germination tests was prepared by dissolution of 2 g yeast extract, 5 g malt extract, and 5 g glucose in 1 L of distilled water. Then, 18 g agar were added (not soluble at room temperature) and the pH of the dispersion was adjusted to 5.5 with hydrochloric acid. After autoclaving the mixture at 121 °C for 40 min, a clear yellow solution was obtained. This solution was cooled to 50–60 °C and approximately 15 mL were distributed into 90 mm petri dishes. This preparation was carried out in a disinfected fume hood and in an aseptic workspace. After one night at room temperature, the plates were stored at 4 °C.

### 2.9 Germination tests

For the germination tests, spore dispersions were typically diluted to $c=10^{3}$ spores/mL. A volume of 100 μL of this dispersion were placed on a petri dish containing the previously prepared YMG medium. Spores were spread on the medium with a sterile cell spreader in an aseptic workspace. After 48–72 h, the mycelium of germinated spores was observable by eye. Germination was calculated on the basis of three petri dishes by the application of Eq. (2).

(2) $$G=\frac{N_{\text{G}}}{N_{\text{t}}\;}\cdot 100\text{ \%}$$

where, G is the germination, $N_{\text{G}}$ is the number of germinated spores, and $N_{\text{t}}$ is the number of spores placed on the medium.

As $N_{\text{G}}$ and $N_{\text{t}}$ are error containing measures, the error ΔG was calculated with Gaussian error propagation, as follows:

(3) $$\Delta G=\pm \sqrt{{\left(\frac{1}{N_{\text{t}}}\cdot \Delta N_{\text{G}}\right)}^{2}+{\left(\frac{N_{\text{G}}}{N_{t}^{2}}\cdot \Delta N_{\text{t}}\right)}^{2}}\cdot 100\text{ \%}$$

where, ΔG is the germination error, $\Delta N_{\text{G}}$ is the standard deviation of the germination, and $\Delta N_{\text{t}}$ is the counting error afflicted to the Neubauer counting chamber (assumed to be 0.3) [35].

### 2.10 Preparation of esca pathogen culture filtrates

For the preparation of culture filtrate of esca pathogens, *Pm. chlamydospora* and *Pa. minimum* were grown in liquid culture medium, which consisted of 4 g yeast extract, 10 g malt extract, and 10 g glucose in 1 L of sterile water. After 7 days, the culture broth was sterile filtered with sterile syringe filters (0.2 μm). The obtained culture filtrate was stored at 4 °C.

### 2.11 Enzymatic degradation of the lignin shell

To degrade the lignin shell, 5 mL of culture filtrate of *Pm. chlamydospora* and *Pa. minimum* were incubated with $5\cdot 10^{5}$ encapsulated spores, respectively. The mixture was filled in a 15 mL centrifuge tube and covered with a Miracloth filter to allow oxygen exchange and also to minimize contamination of other microorganisms. The mixture was continuously shaken for 48 h at 700 rpm and at 25 °C on a thermoshaker. The evaporated solvent was replaced by sterile water. Mycelium of already germinated spores were removed by filtration with a Miracloth filter. Afterward, germination tests were performed. For scanning electron microscope (SEM) measurements, the sample was centrifuged at 9,000 rpm (7,607 rcf) and washed thrice with distilled water to clean spores from culture filtrate residues.

### 2.12 Gel permeation chromatography (GPC) analysis

GPC experiments were performed using an Agilent Technologies 1260 instrument consisting of a pump, autosampler, and column oven. Eluent 80% 0.1 M NaOH and 20% acetonitrile were used in the analysis. A column set consisting of two columns — MCX 105 Å and MCX 103 Å (PSS Standards Service GmbH, Mainz, Germany), both of 300 × 8 mm and 10 μm average particle size — were used at a flow rate of 1.0 mL/min and a column temperature of 30 °C. The injection volume was 25 resp. 10 μL. Detection was accomplished with a UV detector at 270 nm (Agilent Technologies).

Data acquisition and evaluation were performed using the PSS WinGPC UniChrom (PSS-Polymer Standards Service GmbH, Mainz, Germany). Calibration was carried out using polystyrene sulfonate sodium salt standards provided by the PSS-Polymer Standards Service GmbH.

### 2.13 Differential scanning calorimetry analysis

The thermal properties of the shell material were measured by differential scanning calorimetry (DSC) on a METTLER TOLEDO DSC 823e calorimeter. Three scanning cycles of heating–cooling were performed in an N_2_ atmosphere (30 mL/min), with a heating and cooling rate of 10 °C/min. The corresponding lignin material was prepared by adding dropwise 10 mL of a lignosulfonate solution with a concentration of 0.2 wt% to a 0.2 wt% solution of cationic lignin. The mixture was stirred at 1,000 rpm for 15 min. Afterward, the precipitate was centrifuged at 10 ,000 rpm for 10 min, twice. The supernatant was replaced by sterile water. Afterward, the sample was lyophilized for 24 h.

## 3 Results and discussion

To encapsulate the *Trichoderma* spores with the enzyme-degradable lignin shell, a mild technique, which does not damage the cargo, is essential [36]. As the biological cargo is highly sensitive, only gentle mechanical forces, moderate temperatures (0 °C < T < 45 °C), neutral or slightly acidic pH, surfactants and solvents without antimicrobial activity, and compatibility with aseptic work principles are needed. We chose a mild LbL deposition of positively and negatively charged polyelectrolytes in alternating steps onto the charged surface of *Trichoderma* spores as the encapsulation strategy that fulfills all the needs [37].

**Fig. 2:**
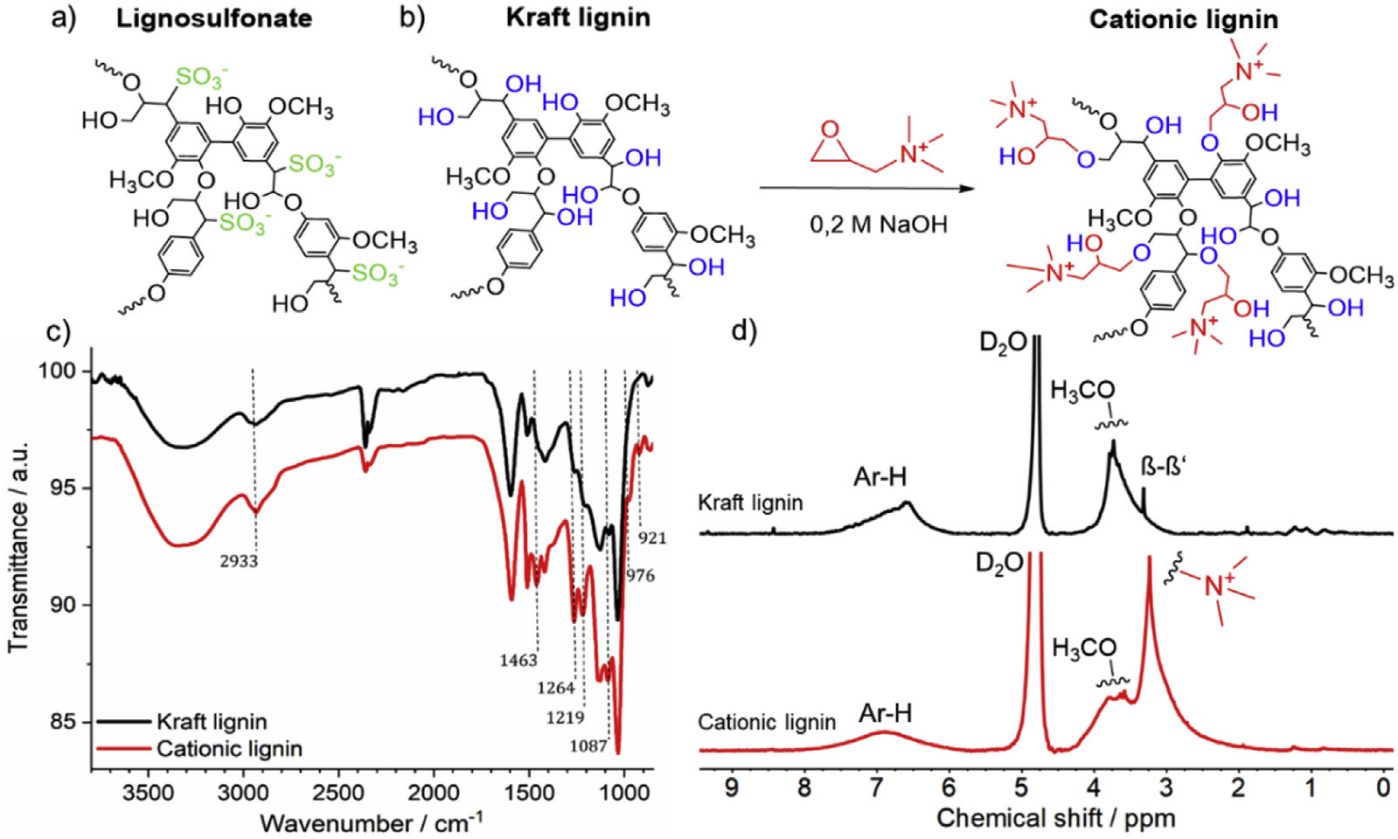
a) Schematic section of a lignosulfonate structure (functionalities and architecture may vary depending on the source and batch). b) Synthesis scheme of cationic lignin from Kraft lignin. c) Comparison of the FT-IR of Kraft lignin and cationic lignin. d) Comparison of the ^1^H NMR of Kraft lignin and cationic lignin.

For the synthesis of an enzyme-degradable lignin polycation, the hydroxyl groups of Kraft lignin were reacted with GTAC [33]. Basic reaction conditions were chosen to reach deprotonation of hydroxyl groups and to increase the solubility in aqueous media. As a result, etherification of hydroxyl groups of Kraft lignin took place and new (2-hydroxypropyl)trimethylammonium groups were introduced. The obtained cationic lignin was soluble at a maximum concentration of 0.25 wt% in sterile water at pH 6 (Figs. 2b and S1).

The presence of new functional groups in the reaction product was verified using FT-IR spectroscopy (Fig. 2c). The spectra of Kraft lignin and cationic lignin show similar bands, which are the typical bands of the Kraft lignin structure (Table S1). The preservation of these bands proves that the aromatic structure was not changed and lignin did not decompose during the reaction. However, changed bands in the FT-IR spectrum prove the successful functionalization of lignin, such as the increased intensity of bands associated with methyl and methylene groups at 2, 933 cm^−1^ [38]. New ether linkages between the lignin structure and the GTAC monomer results in increased intensities for the bands at 1,264 and 1,219 cm^−1^ [39], changing from shoulders in the Kraft lignin spectrum to pronounced bands in the spectrum of cationic lignin. A similar increase is related to the band at 1,087 cm^−1^, which derives from an increased amount of secondary alcohol groups [39]. Furthermore, new bands appear in the cationic lignin spectrum: C–H vibration assigned to trimethylammonium groups at 1,463 cm^−1^ and bands at 976 cm^−1^ and 921 cm^−1^ caused by additional methylene and methine groups are further signs for the successful grafting of GTAC on lignin [33,[40], [41], [42]].

In accordance with the FT-IR spectrum, a comparison of the ^1^H NMR spectra of both compounds shows a new signal at 3.2 ppm, which is associated with the protons of the trimethylammonium group (Fig. 2d) [33]. The signal at 3.3 ppm in the Kraft lignin spectrum was assigned to a ββ' linkage, as shown in Fig. S1. The broad signal from 7.8 to 5.8 ppm, attributed to aromatic protons, remains in both spectra as well as the resonances from 3.0 to 4.0 ppm, attributed to protons in methoxy groups [33]. Further small signals in the range from 0.8 to 2.2 ppm derive from aliphatic chains in the lignin structure. Moreover, the small resonances at 9.3 and 8.4 ppm in the spectrum of Kraft lignin, according to aldehyde and unsubstituted phenolic protons, almost disappear in the spectrum of cationic lignin, which are signs for etherification of alcohol groups and oxidation of the aldehyde groups.

Water-soluble lignin-based polyanions are commercially available in the form of lignosulfonates. Sodium lignin sulfonate, supplied by TCI (Fig. 2a), shows the relevant signals for lignin in the infrared and NMR spectra, as mentioned previously (Figs. S2 and S3) and was water-soluble at a maximum concentration of 15 wt%. Lignosulfonate was used without modification or further purification in the LbL assembly.

**Fig. 3:**
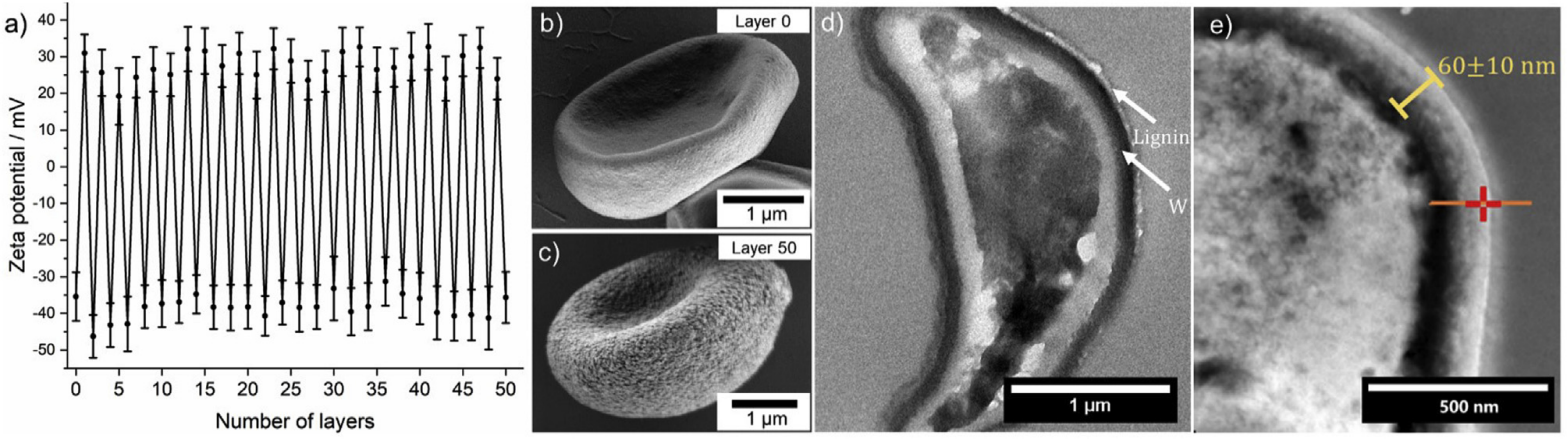
a) Zeta potential during the layer-by-layer encapsulation of *Trichoderma* spores. Error bars indicate the standard deviation of the single measurements. b) SEM image of the spores before the encapsulation. c) SEM image of the spores after 50 steps of the encapsulation. d) TEM image of a histological section of an encapsulated *Trichoderma* spore. W points out the outer cell wall of the spore. e) TEM–EDX measurement of the lignin layer on top of the *Trichoderma* cell wall (20 layers). The orange profile indicates the area of the measurement of the osmium content (Fig. S7).

For the encapsulation process, the spores were dispersed under sterile conditions in sterile water, resulting in a long-term stable dispersion with a negative zeta potential of −35 ± 6 mV. No additional surfactants were needed. However, sedimentation of the spores appeared after several hours, but could be easily prevented by shaking the sample. The zeta potential shifted after each addition step of the respective lignin-based polyelectrolyte, which allowed precise monitoring of the encapsulation process (Fig. 3a).

The adsorption of cationic lignin in the first layer shifted the zeta potential from −35 ± 6 mV by 66 mV to +31 ± 5 mV. The proceeding LbL treatment was characterized by an average shift between −38 ± 4 mV and +28 ± 4 mV. The variation of the number of assembled layers allowed precise control over the shell thickness in the nanoscale. Zeta potential values of at least ±20 mV during all steps delivered adequate stability of all intermediate spore dispersions over the entire process. The high negative zeta potential values in the range of –38 ± 4 mV delivered good long-term colloidal stability of the final spore dispersions and enables the application via the trunk injection technique.

The spore yield over the entire LbL encapsulation consisting of 50 lignin layers was 50%. In each LbL step, 0.5 mL of the respective lignin solution with a concentration of 0.2 wt% were used, which leads to a low amount of lignin needed to perform the entire described LbL process. In a typical batch, 25 mg of the respective lignin electrolyte were sufficient to form a lignin shell consisting of 50 layers for $20\cdot 10^{6}$ spores.

Changes in the surface morphology caused by the lignin deposition are presented in Fig. 3b and c. Lignin builds a continuous shell on top of the cell wall. Notably, cracks can be observed, especially in areas where spores are buckled because of the high vacuum in the SEM (see Figs. S5 and S6 for overview images). Crack formation indicates that the precipitation product of cationic lignin and lignosulfonate forms a rather rigid and therefore brittle coating. This finding is confirmed by the high glass transition temperatures between 97 and 171 °C for lignin derivatives reported in the literature [43] and thermal analysis of the used shell material with glass transition temperatures (*T*_g_) of ca. 70 °C (Fig. S10). A rigid spore coating is beneficial for delivering mechanical stability. In addition, it may form an efficient barrier for nutrients and water and keep the spore in the desired resting state.

In addition, we found that the adsorption of the synthesized cationic lignin is comparable with well-defined polyelectrolytes used in standard LbL assemblies [[44], [45], [46], [47], [48]]. To compare the adsorption behavior of the lignin derivatives with standard LbL electrolytes, we repeated the encapsulation with poly(dimethyldiallylammonium chloride) and poly(sodium 4-styrene sulfonate), resulting in a very similar pattern for the zeta potentials and the surface morphology as for lignin (Fig. S4). Similar adsorption as standard electrolytes enable the synthesized cationic lignin as a sustainable alternative to commonly used synthetic polycations in LbL assemblies for particulate substrates. As bio-based polymers often present native bioactivity in biological systems, cationic lignin could be an attractive building block for other applications in biological sciences as well.

The lignin shell was further investigated by TEM imaging of histological sections of the spores (Fig. 3d). However, in standard electron imaging, the cell wall and the lignin layer cannot be distinguished as their ability to scatter electrons is very similar. Therefore, the spores were stained before the LbL process with osmium tetroxide, a common reagent in dihydroxylation of alkenes. Osmium tetroxide shows a high affinity to membrane lipids because of their high content of olefins. Therefore, osmium tetroxide acts as fixation as well as a staining agent [49]. After several washing steps, aiming at the removal of excess osmium tetroxide, 20 lignin layers were deposited on the stained spores via the LbL process. This procedure secured selective staining of the cell wall and increased the contrast between the spores and the lignin shell in the TEM images. According to Benítez et al. [50] the cell wall of *Trichoderma* spores consists of different glycans, proteins, and melanin, whereby a non-indolic melanin-like polyphenol pigment was found in the outermost layer. Fig. 3d illustrates that the outer wall layer W was significantly stained and therefore appears much darker, which is consistent with the literature [51]. Lignin, which is assigned to the lighter layer on top of the outer cell wall in Fig. 3d, surrounded the entire spore surface. The continuously surrounding lignin layer further proves the successful lignin deposition onto the entire cell wall of the spores. Moreover, EDX was performed to extract the average thickness of the single lignin layers from the TEM images. Fig. S7 reveals a very sharp decrease in osmium content along the orange profile in Fig. 3e. The sharp osmium decrease enabled the precise determination of the beginning of the lignin layer on top of the cell wall. It turned out that the transition from the osmium-rich to the osmium-poor region corresponded to the transition from the stained to the unstained region in the TEM images. With this finding, the average diameter of 20 lignin layers was determined to be 60 ± 10 nm. This leads to an average thickness of 3 ± 0.5 nm for each single lignin layer.

**Fig. 4:**
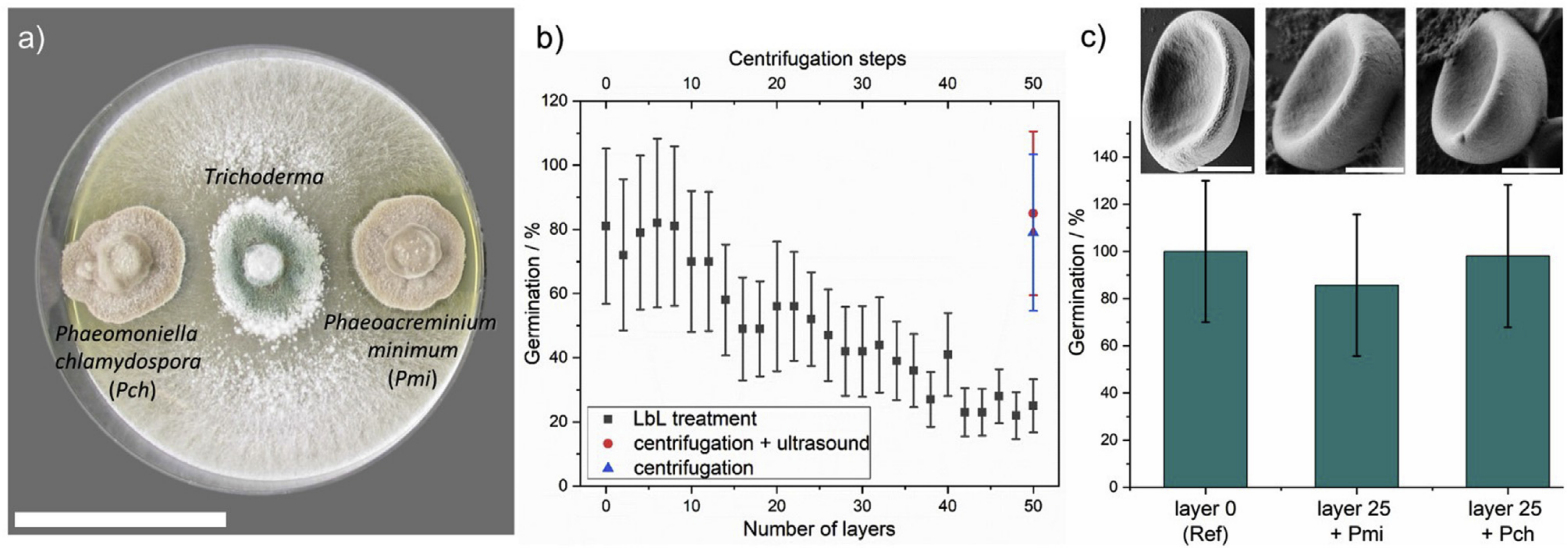
a) Dual culture test of *Trichoderma* against two major esca complex pathogens (*Phaeoacremonium minimum* [*Pmi*] and *Phaeomoniella chlamydospora* [*Pch*]). Scale bar is 5 cm. b) Germination of encapsulated *Trichoderma* spores after 3 days as a function of the capsule thickness. The control samples (red and blue) underwent the LbL procedure, including all centrifugation and redispersion steps. However, lignin adsorption was excluded for the controls. c) Germination of untreated *Trichoderma* (layer 0) and former encapsulated *Trichoderma* (layer 25) after treatment with culture filtrates of *Pmi* and *Pch*. The SEM images show the surface morphology of the corresponding spores. Scale bars are 1 μm.

The most desired ability of *Trichoderma* is its mycoparasitism against pathogenic fungi [3,5,52]. For this reason, the mycoparasitic potential of the strain IBWF 034-05 against the fungi *Pm. chlamydospora* (*Pch*) and *Pa. minimum* (*Pmi*), two major esca pathogens, was tested in a dual culture test (Fig. 4a). After 5 days of inoculation at room temperature, the fungi competed for nutrients in the given habitat. Fig. 4a proves that as soon as *Trichoderma* encountered *Pch* or *Pmi*, the radial growth of both pathogens was inhibited completely. IBWF 034-05 colonized the habitat on the plate medium very fast and proved to be very effective in inhibiting the growth of *Pch* and *Pmi*. In a second stage, *Trichoderma* was able to overgrow both pathogens of the esca complex, penetrated the pathogenic hyphae and supplanted them almost completely from the plate after 10 days (Fig. S9). In the face of these results, the new *T. reesei* strain IBWF 034-05 was identified as a very powerful BCA for a curative treatment against the two major esca pathogens, *Pch* and *Pmi*.

In the next step, the influence of the shell thickness on the germination was investigated. Therefore, spores with different numbers of lignin layers were prepared and germination tests were performed. The data in Fig. 4b clearly reveal the dependence of the germination on the number of lignin layers. However, the initial eight layers did not decrease the germination rate significantly. Most likely, the first layers rather mechanically stabilize the spores, which make them more resistant against the osmotic pressure in distilled water.

A higher number of deposited lignin layers led to a significant and systematic decrease of germination. Spores with 50 lignin layers exhibited germination of 25 ± 8%. However, a further decrease in the germination by an increase in the number of layers is possible.

To control that the centrifugation and redispersion steps during the LbL process did not harm the spores, reference samples were prepared. One of the references underwent just centrifugation and redispersion by vortex, whereas the second reference also underwent additional redispersion in the ultrasonic bath. A third reference was kept untreated (layer 0). Lignin adsorption was excluded for all reference samples. Fig. 4b points out that the spore samples, which were centrifuged or redispersed with the help of ultrasound and/or vortex, did not show a decreased germination compared to the untreated reference sample (layer 0). All three reference samples showed similar germination in the range of 80 ± 20%. Consequently, the LbL approach can be classified as an appropriate tool for the non-damaging encapsulation of the sensitive biological cargo and the control over the germination.

The enzymatic degradation of the lignin shell should result in reactivation of the *Trichoderma* spores and enable an enzyme-responsive delivery. To investigate the degradation of the lignin shell, encapsulated spores with 25 lignin layers were treated with culture filtrate of the two esca pathogens, *Pch* and *Pmi*, respectively. This culture filtrate consisted of a nutrient solution in which the pathogens were grown for 7 days. During their growth, they segregate extracellular compounds such as laccase and other cell wall-degrading enzymes [53,54]. After 1 week of growth, the fungi were removed by sterile filtration. The obtained culture filtrate provided a solution of naturally present enzymes of the esca complex. Therefore, it simulated the esca-infected grapevine environment. Fig. 4c compares the germination of plain spores (layer 0) and lignin-encapsulated spores (layer 25) after 3 days of contact with the culture filtrate. The data are normalized to the reference sample because a filtration step was needed in advance to the germination tests as all samples showed beginning germination after 3 days in the culture filtrate. Even the filtration step went along with unquantifiable spore losses, which reduced the number of spores for all samples, the comparison of encapsulated and untreated spores in Fig. 4c shows that germination of the former encapsulated spores was not decreased anymore. Similar germination indicates that spores are able to recover from the resting state by an enzymatic trigger of esca pathogens.

To confirm the degradation of the lignin shell, we further visualized the spores after the culture filtrate treatment by SEM imaging (Figs. 4c and S8). The images show a significant number of spores with smooth surfaces, indicating that the lignin shell has been removed by the culture filtrate treatment. Besides the spores, some precipitations of culture filtrate were detected, yet they do not adsorb to the spore surface. In comparison with non-encapsulated spores (layer 0; Figs. 3a and S5), the initial surface morphology was restored after the treatment with the culture filtrate, which supports the assumption of successful spore release. The fact that released spores, as well as encapsulated spores, are present might be a result of the sample inhomogeneity during the culture filtrate treatment. Some spores might have been attached to the inner wall of the reaction vessel, because of the coating with surface-active lignin derivatives. Therefore, they did not participate in the lignin degradation process and remain encapsulated. However, the presence of many spores without lignin shells combined with the recovery of the germination provides strong pieces of evidence for the enzymatic degradation of the lignin shell by esca pathogens. By the reactivation of lignin-encapsulated *Trichoderma* spores, the entire concept of a lignin-based delivery system with the controlled enzymatic release by esca pathogens could successfully be realized. The optimum number of lignin layers will be determined by the desired application. A higher number of lignin layers corresponds with more spores in the resting state, which we believe is beneficial for a long-term treatment of the esca disease, but might be varied with the targeted disease.

## 4 Conclusion

In conclusion, we introduced the new strain of *T. reesei* IBWF 034-05 that provides strong antagonism against the major esca pathogens, *Pch* and *Pmi*, and offers great potential as a curative BCA. We encapsulated the *Trichoderma* spores in lignin-based polyelectrolyte shells via the LbL technique creating a surfactant-free, self-stabilizing spore dispersion. The lignin shell transfers the spores in a resting state without inflicting damage. The resulting spore dispersion is colloidally stable over several months and applicable via trunk injection. Once injected, encapsulated spores fulfill the concept of a Trojan horse. If the plant is infected by esca pathogens, lignin-degrading enzymes, produced by the pathogens itself, degrade the lignin shell and trigger the germination process. Germinated *T. reesei* is able to parasitize the fungal pathogens and to supplant them from the habitat. At the same time, *T. reesei* strengthens plants against future infections. This concept enables the application of *T. reesei* IBWF 034-05 for protective as well as curative treatments of esca, one of the most infective grapevine trunk diseases worldwide.

## Funding

This work was supported by the 10.13039/501100001663Volkswagen Stiftung (Experiment ‘NanoProtect’).

## Declaration of competing interest

The authors declare that they have no known competing financial interests or personal relationships that could have appeared to influence the work reported in this paper.

## Data availability

Experimental data will be made available on request.

## Author contributions

**S. Peil, S.J. Beckers:** Investigation, Data curation, Formal analysis, Validation, Writing - original draft, Writing - review & editing.

**J. Fischer:** Funding acquisition, Project administration, Investigation, Writing – review & editing.

**F.R. Wurm:** Funding acquisition, Project administration, Resources, Software, Supervision, Validation, Writing – review & editing.
