# Supplementary material for: Biodegradable, lignin-based encapsulation enables delivery of Trichoderma reesei with programmed enzymatic release against grapevine trunk diseases
Source: Mater Today Bio. 2020 Jun 20;7:100061. doi: 10.1016/j.mtbio.2020.100061 (PMC7327927; doi:10.1016/j.mtbio.2020.100061)
Supplement: Multimedia component 1 [file mmc1.doc]

Supplementary data

Biodegradable, lignin-based encapsulation enables delivery of *Trichoderma* *reesei* with programmed enzymatic release against grapevine trunk diseases

*Stefan Peil, Sebastian J. Beckers, Jochen Fischer, and Frederik R. Wurm**

^a^Max-Planck-Insitute for Polymer Research, Ackermannweg 10, 55128 Mainz, Germany; wurm@mpip-mainz.mpg.de

^b^Institute for Biotechnology and Drug Research, Erwin-Schrödinger-Str. 56, 67663 Kaiserslautern, Germany

Supplementary spectra and images


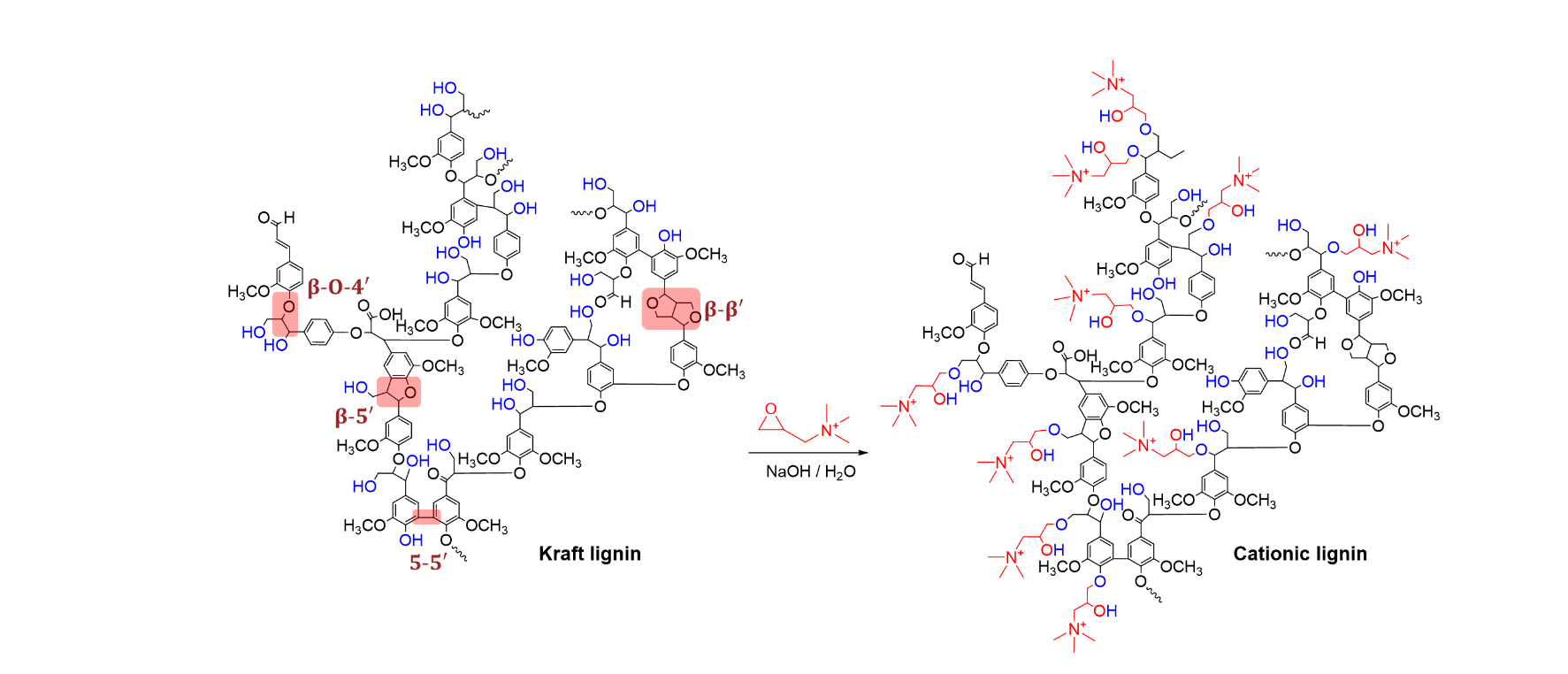


Figure S1: Modification of Kraft lignin with GTAC. Common linkages in the Kraft lignin structure are highlighted. Depending on the source, functionalities and architecture may vary.


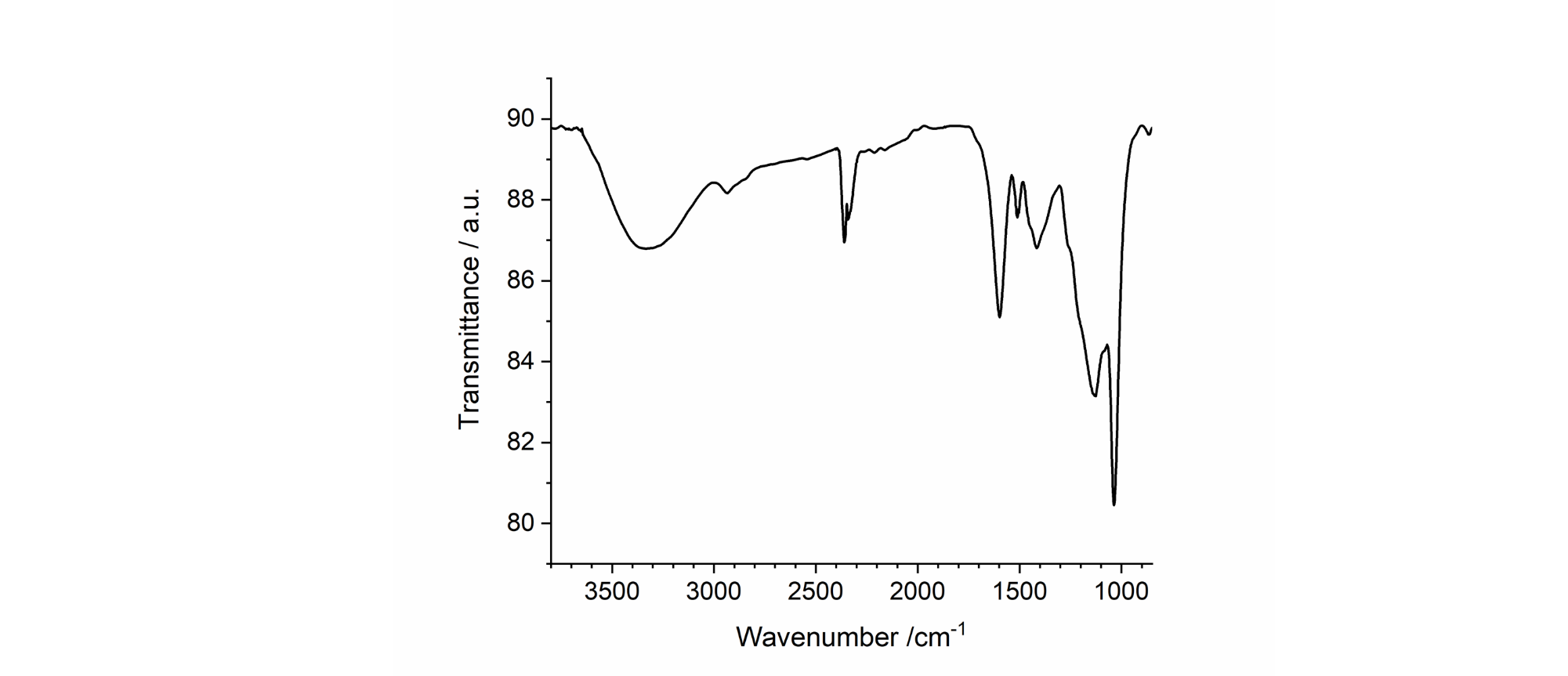


Figure S2: FT-IR spectrum of sodium ligninsulfonate (supplied by TCI, Lot: V5VJF-NC).


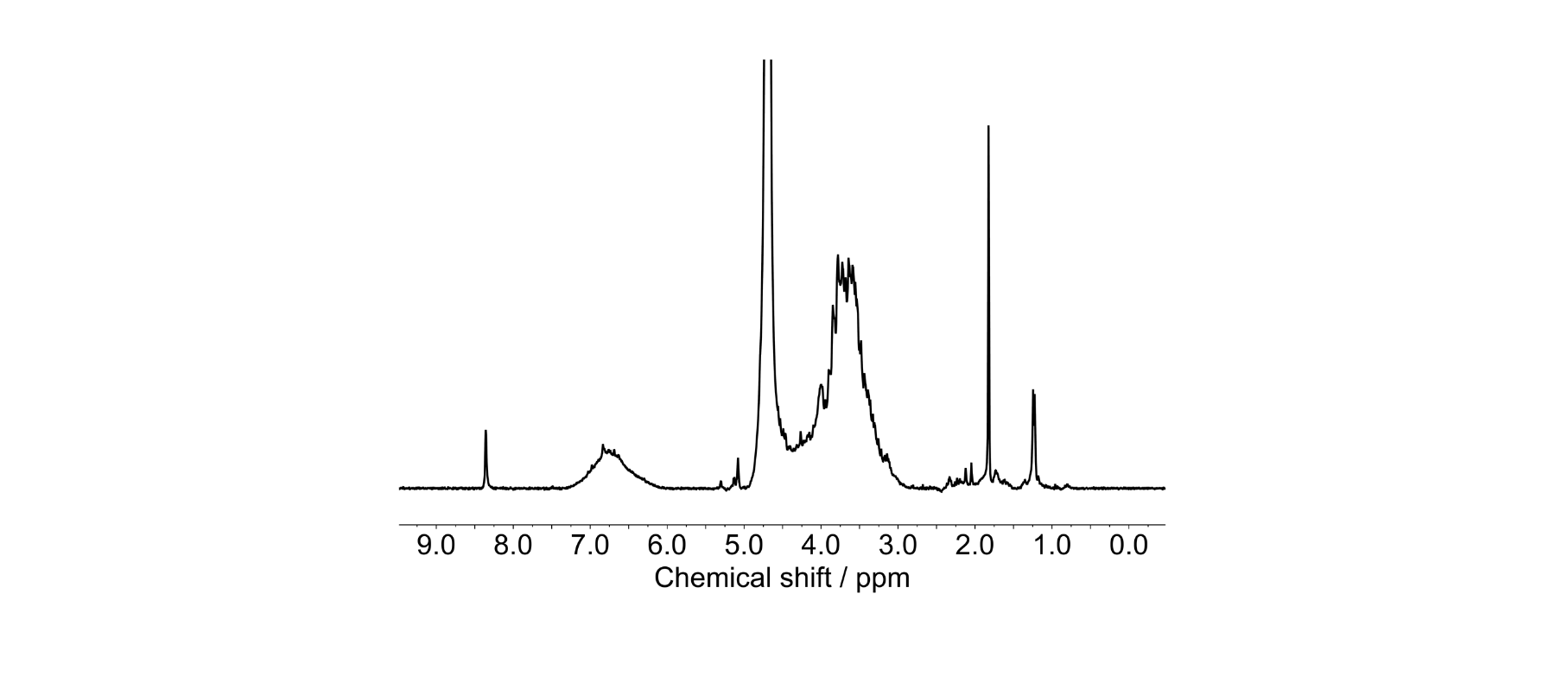


Figure S3: ^1^H-NMR spectrum of sodium ligninsulfonate (supplied by TCI, Lot: V5VJF-NC). The solvent was D_2_O.

Table S1: FT-IR band list of the Kraft lignin structure, cationic lignin, and sodium lignin sulfonate.[1-9]

| Band position / cm^-1^ | | | Assignment |
| --- | --- | --- | --- |
| Kraft lignin (Sigma) | Cationic lignin | Sodium lignin  sulfonate (TCI) |
| 3319 | 3350 | 3332 | O-H stretching (hydrogen bonded) |
| 2934 | 2933 | 2938 | C-H stretching, aliphatic |
| 2360, 2341 | 2358, 2344 | 2360, 2341 | O=C=O stretching (not assigned with lignin structure) |
| 1597, 1510 | 1592, 1510 | 1597, 1510 | C=C skeletal vibration |
| - | 1463 | - | C-H of GTAC unit (CH_3_) |
| 1416 | 1421 | 1415 | C-H in-plane deformation  with aromatic ring stretching |
| 1261, 1208 | 1264, 1219 | - | C-O of guaiacyl unit (alkyl aryl ether) |
| 1127 | 1127 | 1126 | C-H of guaiacyl and syringyl unit |
| 1082 | 1087 | - | C-O secondary alcohols |
| 1033 | 1032 | 1036 | C-O of primary alcohol, guaiacyl C-H |
| - | 976 | - | C-H of GTAC unit (CH_2_) |
| - | 921 | - | C-H of GTAC unit (CH_3_) |
| 876 | 862 | 865 | C-H out of plane aromatic ring |


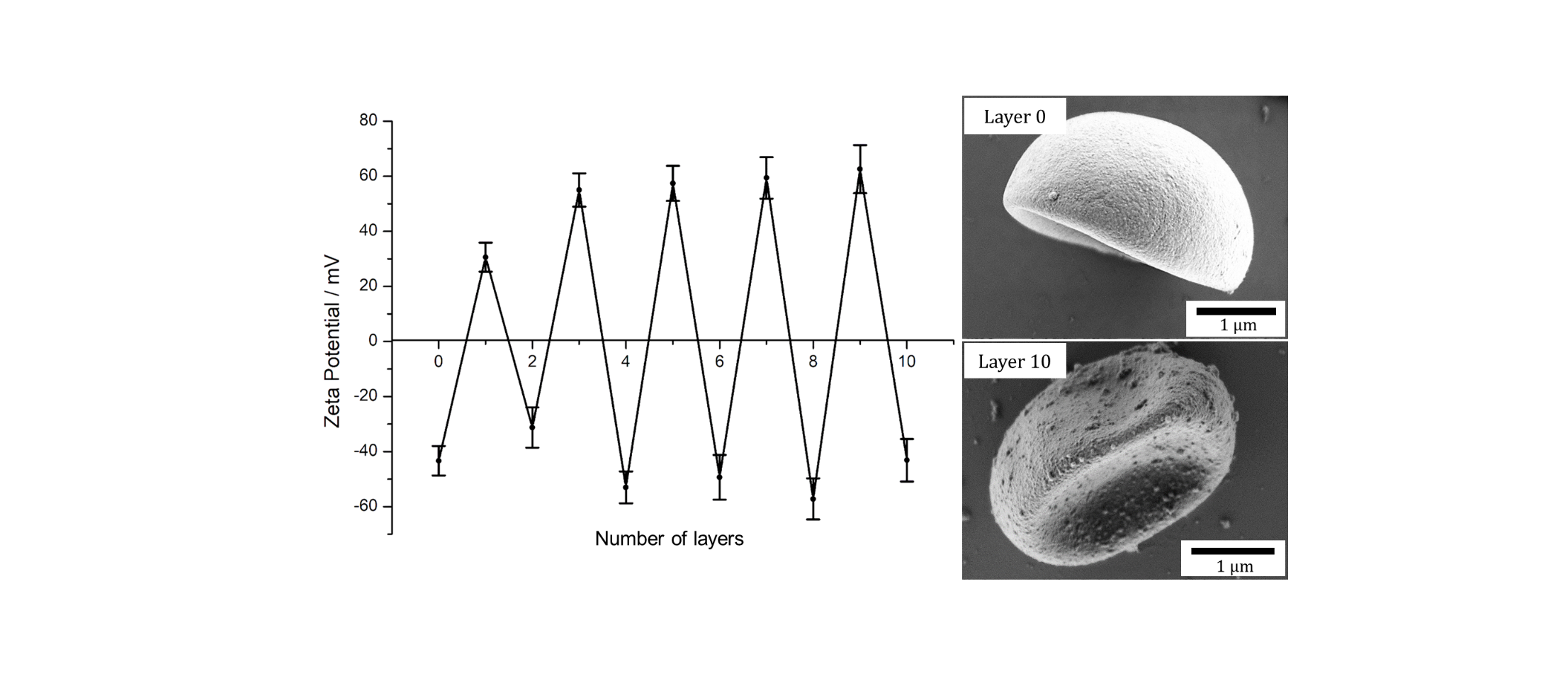


Figure S4: Layer-by-layer encapsulation of Trichoderma spores with conventional polyelectrolytes (PDADMAC/PSS): left: Zeta potential during the layer-by-layer encapsulation of *Trichoderma* spores (strain IBWF-034-05) and right: SEM images of the spores before and after encapsulation.


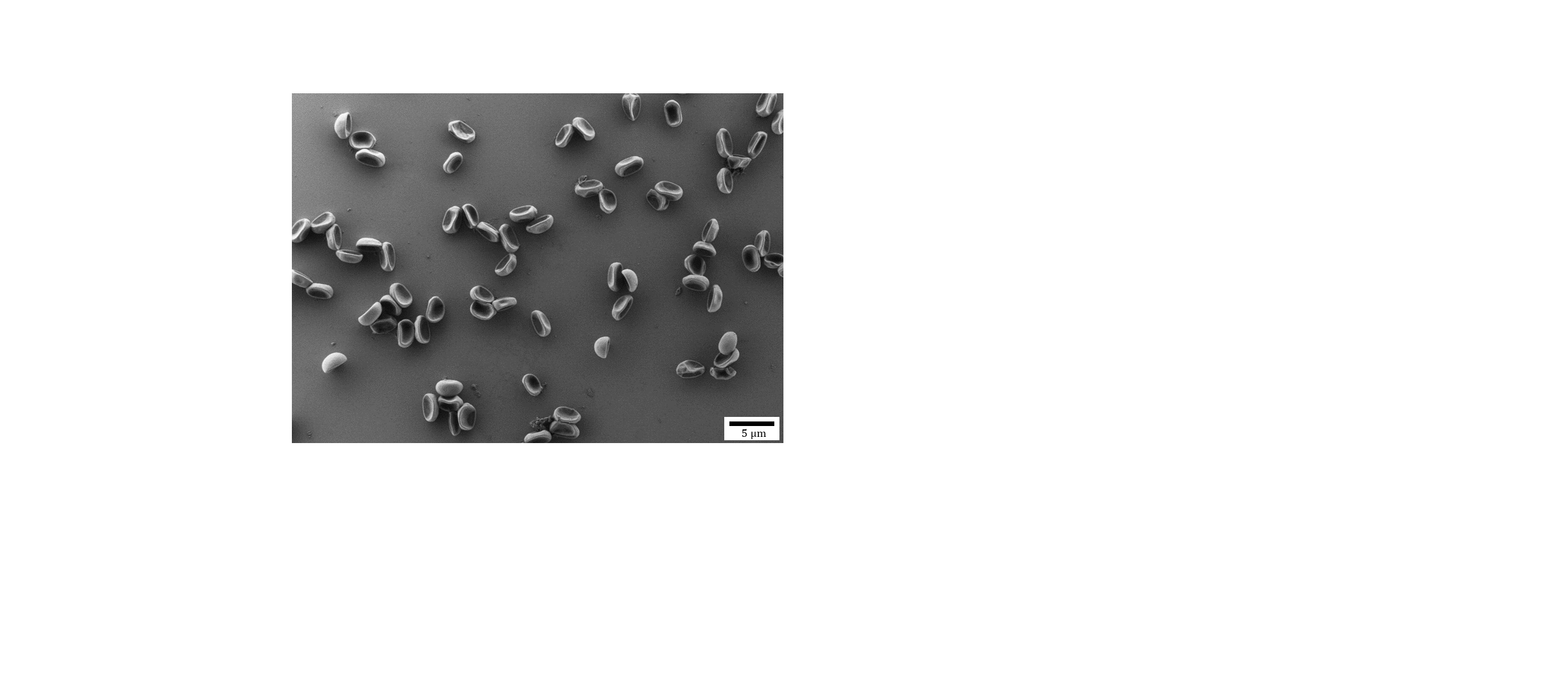


Figure S5: Overview SEM image of *Trichoderma reesei* strain IBWF 034-05.


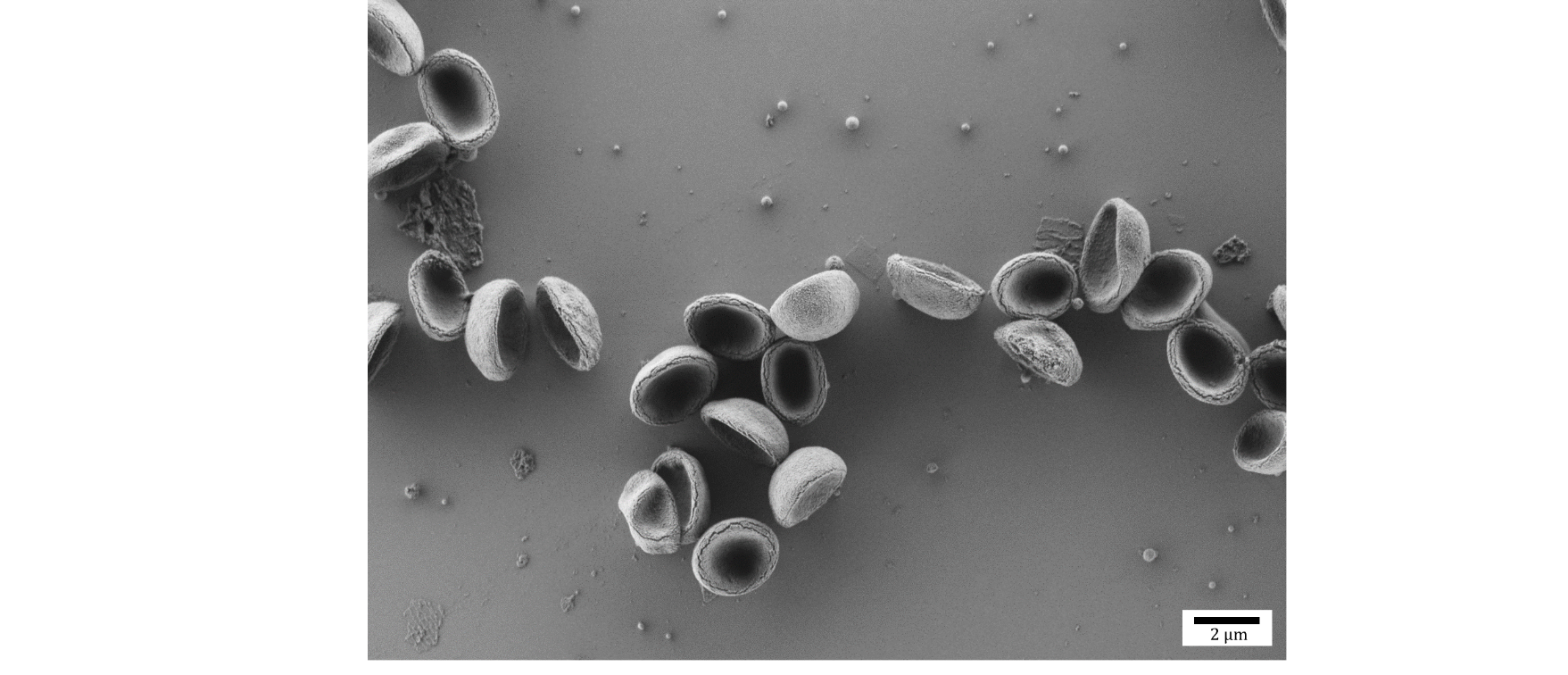


Figure S6: Overview SEM image of lignin-encapsulated *Trichoderma reesei* strain IBWF 034-05 (layer 20).


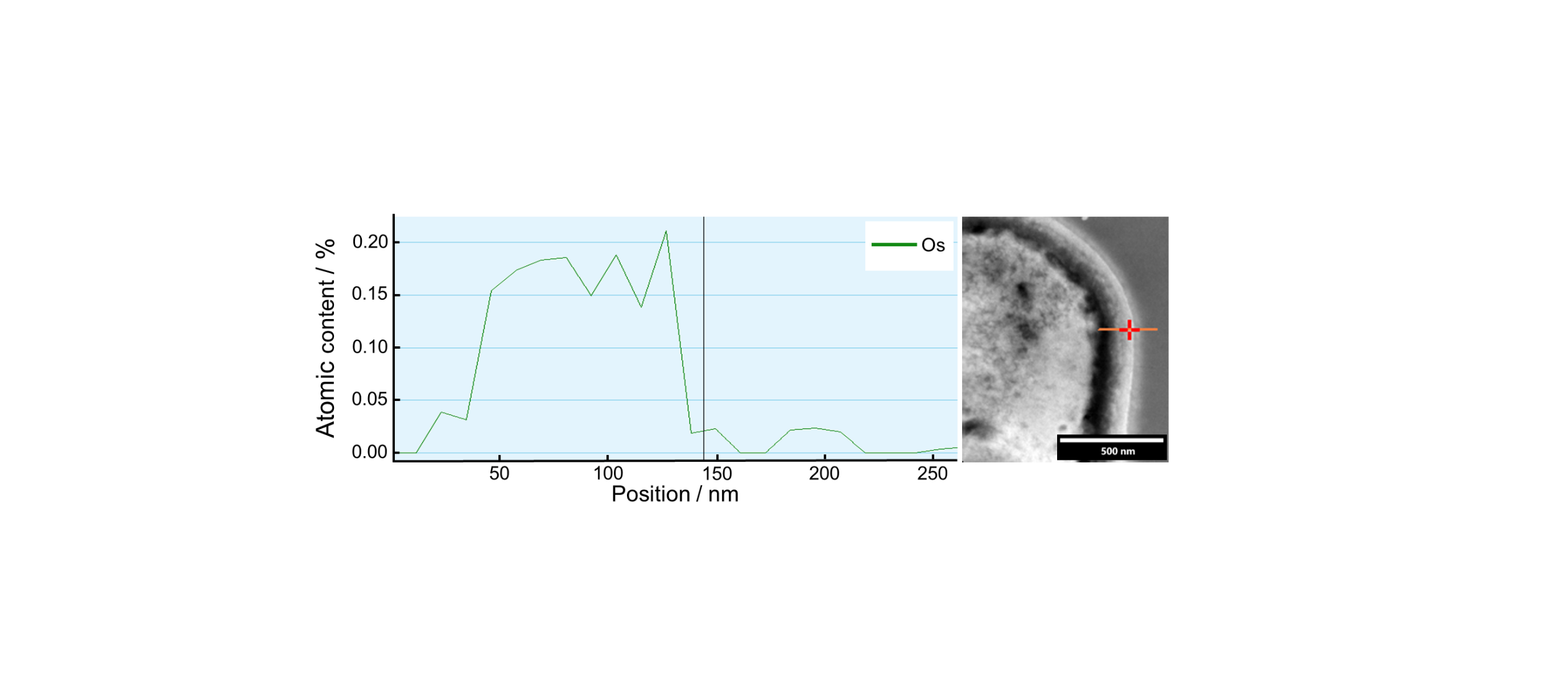


Figure S7: EDX profile along the cell wall of a lignin encapsulated *Trichoderma* spore. Osmium counts as a function of position along the orange bar in the TEM image on the right.


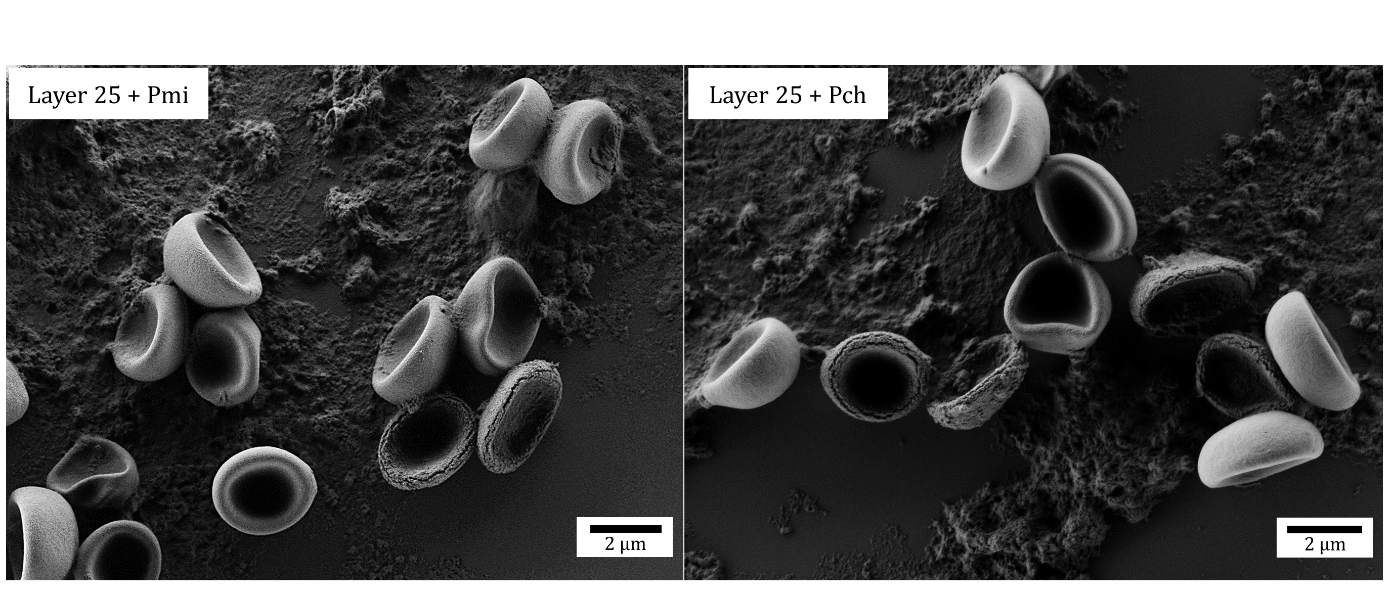


Figure S8: Overview SEM image of lignin-encapsulated *Trichoderma reesei* after treatment with culture filtrate of *Pch* and *Pmi*.


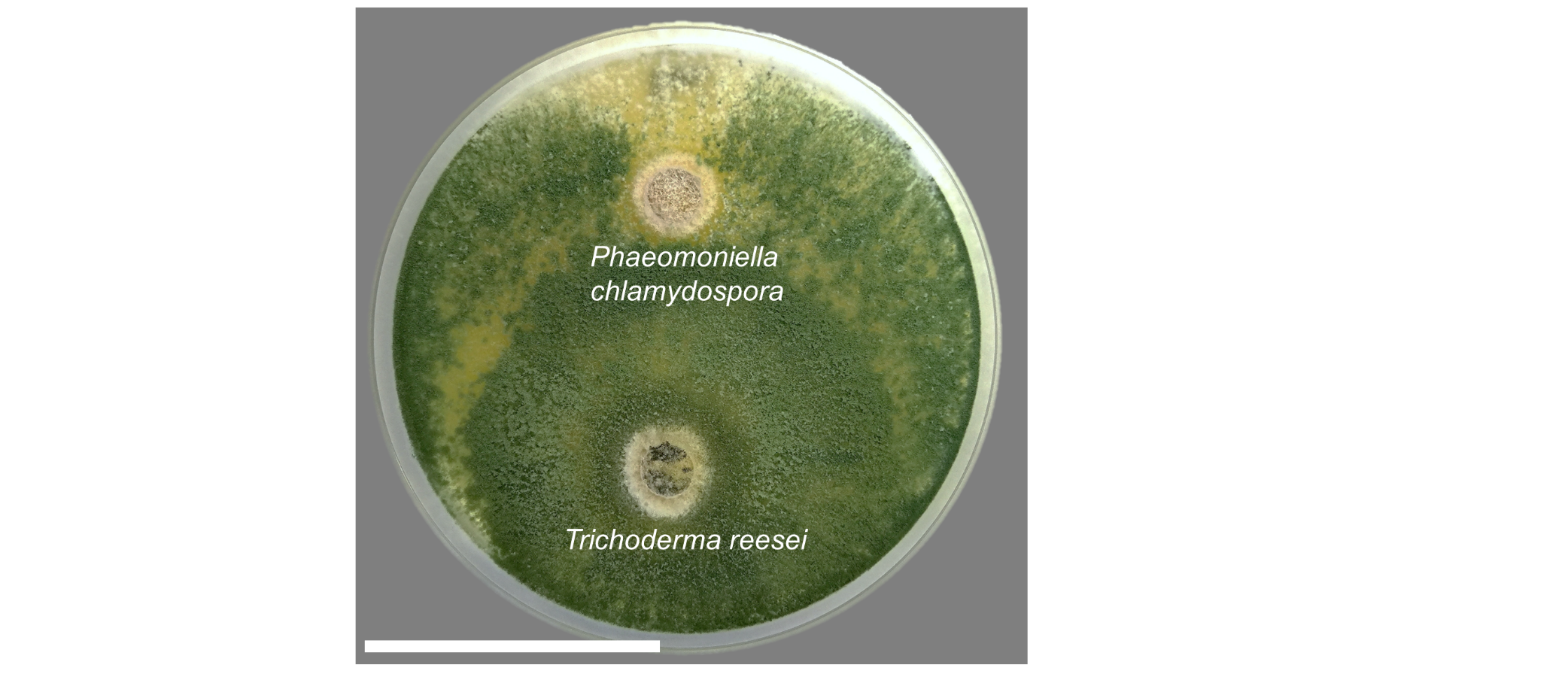


Figure S9: Dual culture test of the *Trichoderma* *reesei* strain IBWF 034-05 against *Phaeomoniella* *chlamydospora* after 10 days. Scale bar is 5 cm.


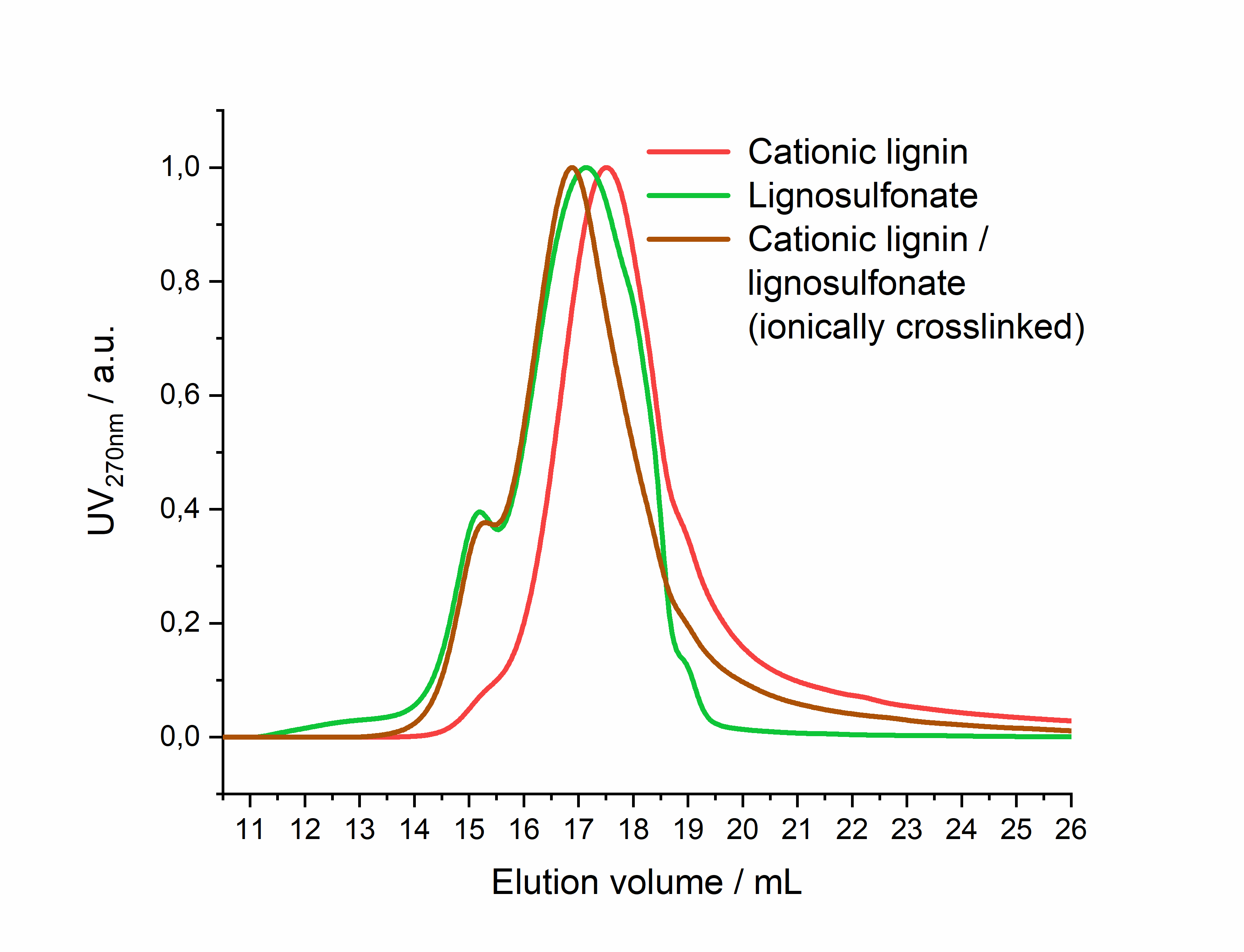


Figure S9: GPC elugrams of cationic lignin, lignosulfonate, and ionically crosslinked lignin consisting of cationic lignin and lignosulfonate (eluent: 80% 0.1 M NaOH, 20% acetonitrile). (evaluation vs Polystyrene sulfonate standards: *M*_w_= 2,400 g/mol for cationic lignin,
*M*_w_= 9,700 g/mol for lignosulfonate, *M*_w_ = 7,900 g/mol for the mixture of cationic lignin/lignosulfonate).


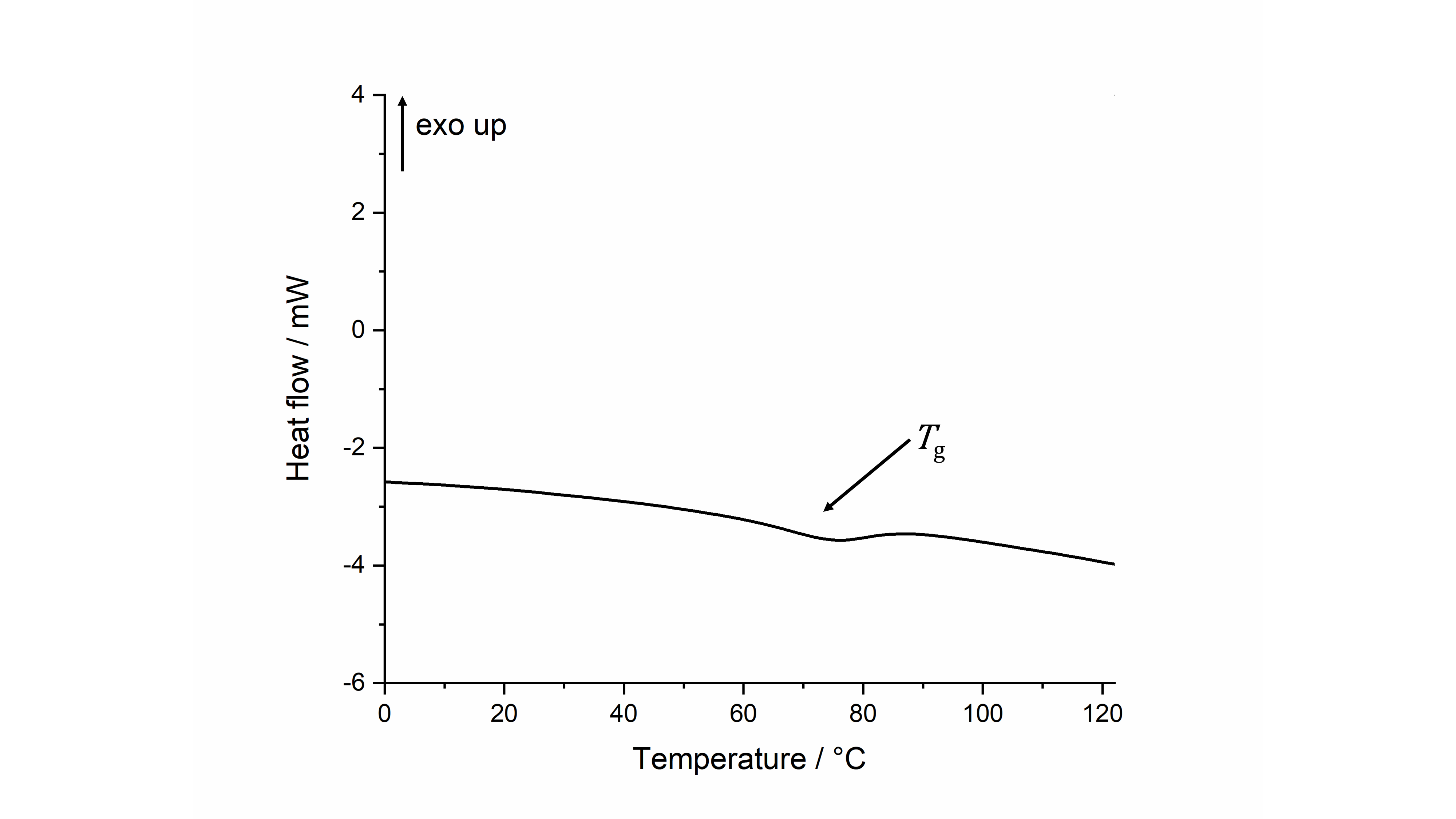


Figure S10: DSC heating curve of the ionically crosslinked lignin shell material (first heating, 10 K/min), *T*_g_ = ca. 70°C.

**References**

1. Cho, J.*, et al.*, *Biomacromolecules* (2006) **7** (10), 2845-2855

2. Ebringerová, A.*, et al.*, *Carbohydrate Polymers* (1994) **24** (4), 301-308

3. Kubo, S., and Kadla, J. F., *Biomacromolecules* (2005) **6** (5), 2815-2821

4. LISPERGUER, J.*, et al.*, (2009) **54** (4)

5. Nada, A.-A. M. A.*, et al.*, *Polymer Degradation and Stability* (1998) **62** (1), 157–163

6. Carbon dioxide.

7. Pandey, K. K., (1999) **71** (12), 1969-1975

8. Popescu, C.-M.*, et al.*, *Applied spectroscopy* (2007) **61** (11), 1168–1177

9. Ruihua, H.*, et al.*, *Journal of Materials Science* (2012) **47** (2), 845–851
